# Toll-like receptor 4 (TLR4): new insight immune and aging

**DOI:** 10.1186/s12979-023-00383-3

**Published:** 2023-11-24

**Authors:** Hyo-Jin Kim, Hyemin Kim, Jeong-Hyung Lee, Cheol Hwangbo

**Affiliations:** 1 Division of Life Science, College of Natural Sciences, Gyeongsang National University, Jinju, 52828 Republic of Korea; 2 Division of Applied Life Science (BK21 Four), Research Institute of Life Sciences, Gyeongsang National University, Jinju, 52828 Republic of Korea; 3 Department of Biochemistry (BK21 Four), College of Natural Sciences, Kangwon National University, Chuncheon, 24414 Republic of Korea

**Keywords:** TLR4, Age-related disease, Cellular senescence, Aging

## Abstract

TLR4, a transmembrane receptor, plays a central role in the innate immune response. TLR4 not only engages with exogenous ligands at the cellular membrane’s surface but also interacts with intracellular ligands, initiating intricate intracellular signaling cascades. Through MyD88, an adaptor protein, TLR4 activates transcription factors NF-κB and AP-1, thereby facilitating the upregulation of pro-inflammatory cytokines. Another adapter protein linked to TLR4, known as TRIF, autonomously propagates signaling pathways, resulting in heightened interferon expression. Recently, TLR4 has garnered attention as a significant factor in the regulation of symptoms in aging-related disorders. The persistent inflammatory response triggered by TLR4 contributes to the onset and exacerbation of these disorders. In addition, alterations in TLR4 expression levels play a pivotal role in modifying the manifestations of age-related diseases. In this review, we aim to consolidate the impact of TLR4 on cellular senescence and aging-related ailments, highlighting the potential of TLR4 as a novel therapeutic target that extends beyond immune responses.

## Background

 Toll-like receptor (TLR) is a type of pattern recognition receptor (PRR) that plays a crucial role in the immune system [1]. PRRs, predominantly expressed by innate immune cells such as dendritic cells, macrophages, monocytes, neutrophils, and epithelial cells, serve as sentinels of the body’s defenses [2]. They become activated upon detecting pathogen-associated molecular patterns (PAMPs), which are molecular signatures unique to external pathogens and distinct from host components, as well as damage-associated molecular patterns (DAMPs), encompassing molecules like heat shock proteins (HSPs) and plasma membrane components released due to cellular damage or death [3]. PRR is a major factor in innate immunity and also plays a role initiating adaptive immunity through induce the maturation of dendritic cells and the release of inflammatory cytokines [4, 5].

TLR is the first identified PRR that recognizes a wide range of pathogens [6, 7]. These receptors are ubiquitously expressed in various innate immune cells, including macrophages, neutrophils, dendritic cells (DCs), natural killer (NK) cells, mast cells, eosinophils, and basophils, all instrumental in mounting innate immune responses against invading pathogens [8]. Structurally, TLRs are transmembrane proteins featuring a leucine-rich extracellular domain responsible for ligand binding and a cytosolic Toll-IL-1 receptor (TIR) domain that induces intracellular signaling [6]. Upon activation, TLRs enlist adaptor proteins, such as myeloid differentiation primary response protein 88 (MyD88), Toll/interleukin-1 receptor domain-containing adapter protein (TIRAP), TIRAP-inducing IFN-β (TRIF), and TRIF-related adaptor molecule (TRAM), alongside protein kinases including inhibitor of NF-κB kinase (IKKi), interleukin-1 receptor-associated kinases (IRAKs) 1 and 4, and Tank-binding kinase (TBK)1, all housed within the cytoplasm of immune cells, to propagate ligand-induced signal transduction cascades [9]. Subsequent activation of downstream proteins leads to cytokine production, proliferation, and survival, with some signals leading to greater adaptive immunity [10].

TLR activation serves as a defense mechanism for the host against infections and tissue damage, initiating a signaling cascade that leads to the secretion of various inflammatory cytokines and the activation of immune cells [11, 12]. Notably, TLR4, a pivotal member of the innate immune response, becomes activated by diverse ligands classified as PAMPs and DAMPs, utilizing both MyD88-dependent and independent pathways [13, 14]. However, excessive TLR4 activation disrupts immune homeostasis by sustaining pro-inflammatory cytokine and chemokine production, thus contributing to the onset and progression of various diseases, including Alzheimer’s disease, cancer, osteoarthritis, and sepsis [15–18].

Aging is a physiological and pathological process that leads to progressive organ damage, extending beyond the cellular level, disrupting organismal homeostasis, and ultimately culminating in mortality [19]. The aging process significantly impacts the immune system, fostering a bidirectional influence termed ‘immunosenescence’ [20, 21]. Cellular senescence triggers the release of senescence-associated secretory phenotype (SASP), which can induce inflammation, subsequently promoting the generation of damage-associated molecular patterns (DAMPs), and escalating the exposure and circulation of externally infiltrated pathogen-associated molecular patterns (PAMPs) due to barrier deterioration [19, 22, 23]. Diverse factors heightened by the aging process result in aberrant immune system regulation through pattern recognition receptors (PRRs), such as Toll-like receptors (TLRs), consequently affecting cardiovascular, metabolic, and age-related degenerative diseases [24]. In this review, we delineate the role of TLR4, a pivotal component of the immune system, and its association with aging-related diseases, thereby shedding light on the significance of TLR4 signaling in disease research.

## TLR family

TLR expression is widespread among immune cells, encompassing innate immune cells such as DCs, macrophages, NK cells, as well as adaptive immune cells, including T cells and B cells. Furthermore, TLRs are present in non-immune cell types, notably in epithelial and endothelial cells [8]. TLRs are classified into two subfamilies based on their localization: cell surface TLRs include TLR1, TLR2, TLR4, TLR5, TLR6, and TLR10, whereas intracellular TLRs include TLR3, TLR7, TLR8, TLR9, TLR11, TLR12, and TLR13 [25]. Cell surface TLRs mainly recognize microbial membrane molecules and induce inflammatory reactions [25]. On the other hand, intracellular TLRs play a role in inducing inflammatory responses by recognizing nucleic acids, mainly derived from bacteria or viruses. However, incorrect recognition of self-nucleic acids can lead to autoimmune diseases [26].

## TLRs and ligand

TLR1 forms a heterodimer with TLR2, allowing it to recognize triacyl lipopeptides from the bacterial outer membrane. TLR2, in turn, engages in heterodimerization with TLR6, enabling it to distinguish diacyl lipopeptides as ligands, setting it apart from TLR1 [27]. When ligands interact with these two heterodimers, they adopt a ‘m’-shaped conformation, enhancing receptor stability and activating downstream signaling cascades [28, 29]. TLR5 recognizes a specific protein present in the flagella filaments of motile bacteria, which is conserved in all bacteria, making it a straightforward process for TLR5 to identify bacteria [30, 31]. While the exact ligand for TLR10 remains elusive, in silico analyses have suggested the possibility of its recognition of lipopeptides, similar to the ligands of the TLR2 complex [32]. Unlike other TLR families, TLR10 induces an anti-inflammatory response, as observed through reduced cytokine expression and release in human cells overexpressing TLR10 and activated through antibody-mediated binding. The exact mechanism of TLR10 is not yet known, but the activation of this receptor has been shown to inhibit the nuclear factor kappa B (NF-κB), mitogen-activated protein kinase (MAPK), and Akt signaling pathways stimulated by other TLRs [33].

TLR3 is one of the intracellular TLR family members and recognizes double-stranded RNA of viruses as a ligand [34]. TLR3 exclusively associates with the adaptor protein TRIF, triggering an antiviral response characterized by heightened activity of interferon regulatory factor (IRF) 3 and the secretion of type I interferons (IFNs) [35]. Activation of TLR3 not only provides protection against atherosclerosis, brain ischemia, and reactive astrogliosis but also promotes hair follicle regeneration in skin wounds [36–38].

The genes of TLR7 and TLR8 are closely located on the X chromosome and recognize single-stranded RNA from viruses as ligands [39, 40]. TLR7 is primarily expressed in the lung, placenta, and spleen and is also associated with autoimmune disorders such as lupus [41, 42]. TLR8 is predominantly expressed in the lung and peripheral blood leukocytes and plays a role in the production of inflammatory factors related to tumor development through the activation of dendritic cells [43]. Both receptors activate transcriptional activity of NF-κB through MyD88 binding, thereby regulating antiviral responses via cytokine production [43].

TLR9 recognizes DNA with unmethylated CpG motifs from bacteria or viruses, making it a vital receptor in immune cells such as dendritic cells, macrophages, B lymphocytes, monocytes, and NK cells [44]. TLR9 is a crucial factor in controlling autoimmune diseases, and active research on TLR9 agonists and antagonists to improve autoimmune inflammation is ongoing [45, 46].

TLR11 recognizes bacterial flagellin and eukaryotic profilin in microorganisms, leading to increased secretion of tumor necrosis factor (TNF)-α, IL-12, and IFN-γ. These cytokines perform important functions in both the innate and adaptive immunity of the host [47, 48]. When TLR11 signaling is stimulated in response to the invasion of Toxoplasma gondii or uropathogenic Escherichia coli, NF-κB is activated, leading to increased expression of IL-12 and chemokines and inducing the maturation of immune cells [47, 49].

TLR12 also recognizes Toxoplasma gondii profilin, activates signaling, promotes the production of IFN-γ in NK cells through the secretion of IL-12 and IFN-α in dendritic cells, thereby bolstering the host’s immune system [50, 51].

TLR13, which is not present in humans, has been identified as an endosomal receptor that recognizes the specific motif of bacterial 23 S rRNA [52–54]. TLR13 induces an innate immune response through the activation of NF-κB via the MyD88 pathway [55].

## TLR4 signaling pathway

**Fig. 1:**
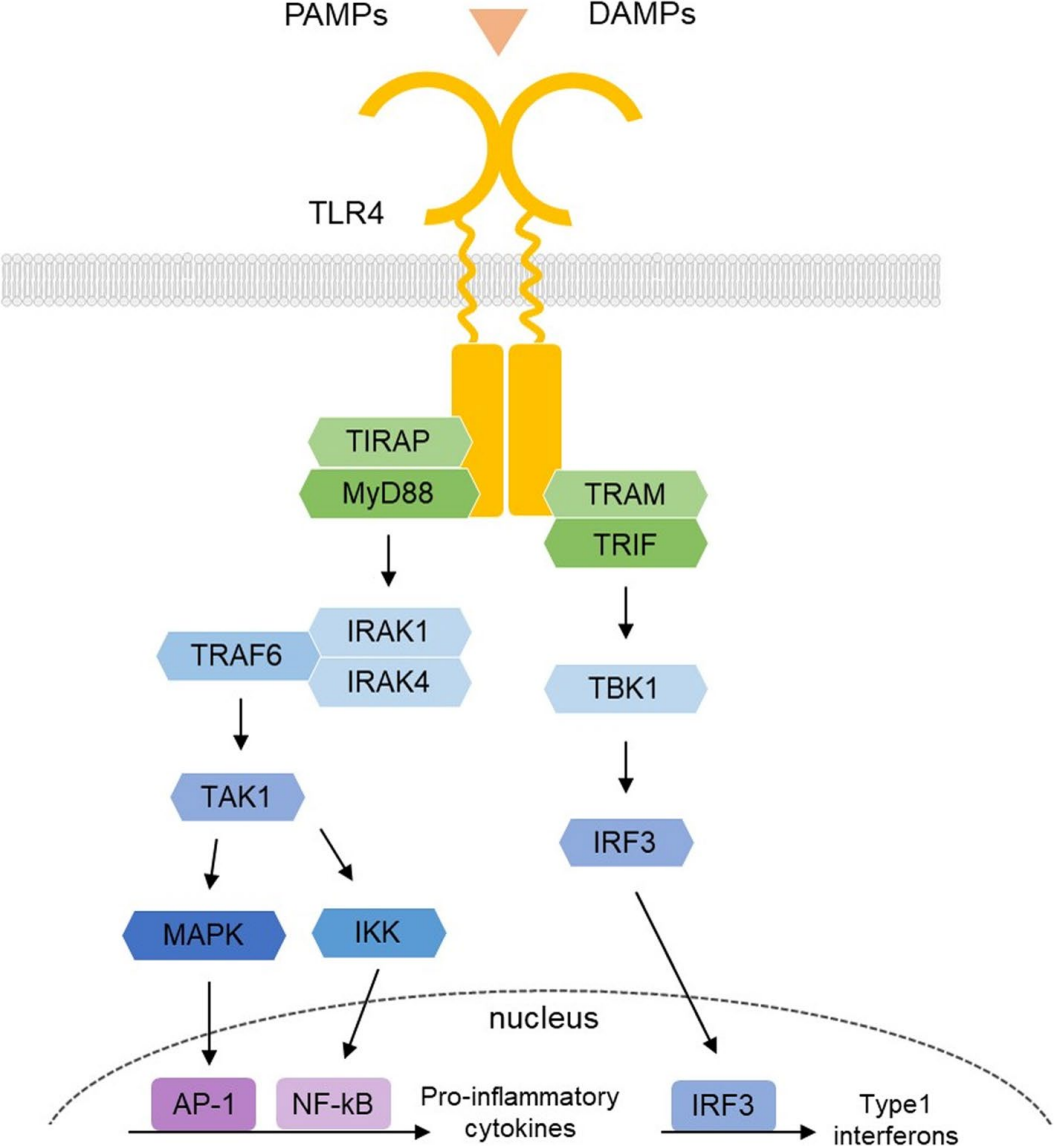
TLR4 signaling pathways: MyD88-dependent and independent downstream signaling. MyD88-dependent signaling produces pro-inflammatory cytokines through IRAK/TRAF6/TAK1 and MAPK an NF-κB activation. MyD88-independent signaling produces type I IFNs through TRIF/TRAM/TBK1 and IRF3 activation

 TLR4 is a transmembrane protein composed of three domains: the extracellular domain, the transmembrane domain, and the cytoplasmic domain. The extracellular domain contains leucine-rich repeat (LRR) motifs, each consisting of 20–29 residues, providing specific sites for ligand interaction [56]. Lipopolysaccharide (LPS), derived from gram-negative bacteria, serves as a prominent representative ligand for TLR4 [57, 58]. Since LPS cannot interact directly with TLR4, it binds to TLR4 via the adapter protein myeloid differentiation factor-2 (MD-2) [59]. Subsequently, the LPS/TLR4/MD-2 complex forms a dimer, initiating intracellular signaling [60]. Beyond LPS, TLR4 is activated by diverse PAMPs sourced from bacteria, viruses, and fungi [61–64]. DAMPs derived from cells can include extracellular matrix molecules [65–68] and intracellular factors, including DNA-binding proteins like high-mobility group box 1 (HMGB1) and cellular HSPs [69–74]. Although the exact binding mechanisms to TLR4 have not been identified for some of the DAMPs, it has been clarified that they induce an immune response through TLR4 signaling (Fig. 1).

 The intracellular signal of TLR4 is transmitted through two separate pathways: MyD88-dependent and independent signaling, which work competitively with each other [75]. The intracellular TIR domain of TLR4 facilitates the binding of various adapter proteins [76]. Dimerization of the TIR domain leads to the recruitment of MyD88 and TIRAP (also known as MyD88-adaptor-like (MAL)) adapter proteins, promoting signaling via IRAKs through their phosphorylation. Activated IRAK binds to TAK1, facilitated by the adapter protein TNF-receptor-associated factor 6 (TRAF6) and TAK1-binding proteins 2 and 3 (TAB2 and TAB3), enhancing kinase activity. TAK1 subsequently activates MAPKs, including JNK, ERK1/2, p38, and the IkB kinase complex (IKK), leading to the activation of pivotal transcription factors such as NF-κB and activator protein-1 (AP-1). These transcription factors promote the production of inflammatory cytokines [77, 78]. Pharmacological interventions targeted at MyD88 show promise as a therapeutic approach for chronic inflammatory diseases and have demonstrated efficacy in ameliorating acute myocardial infarction (AMI) and tumor growth. Moreover, the inhibition of MyD88 exhibits antiviral effects, indicating its potential as a therapeutic target for lung pathologies resulting from exaggerated immune responses and cytokine secretion triggered by COVID-19 infection [79].

MyD88-independent signaling takes place within endosomes following the internalization of the TLR4-ligand complex. It is activated through the coupling of the TIR domain with TRIF and TRAM. This signaling pathway activates TBK1 and IKKε via TRAF3, facilitating the transport of the transcription factor IRF3 into the nucleus. IRF3 initiates the production of type I IFNs [80, 81].

## TLR4 and inflammaging

Innate immune cells hold a significant role in the sustenance of chronic and low-grade pro-inflammatory elements, denoted as ‘inflammaging,‘ throughout the aging process. TLR4-mediated TNF and IL6, both being pro-inflammatory factors, assume pivotal roles in driving the inflammaging phenomenon [82]. TLR4-mediated IL-8 demonstrates an increase in monocytes obtained from aged individuals in comparison to their younger counterparts [83, 84]. Aging-induced activation of reactive oxygen species (ROS) triggers TLR4 signaling, resulting in a subsequent surge of cytokines and chemokines, thereby establishing a feedback loop that further potentiates ROS activity [85]. This sequence promotes immune cell senescence, ultimately influencing organismal aging [86].

Excessive activation of TLR4 plays a significant role in driving the persistent activation of the innate immune system within skeletal muscle, ultimately culminating in the initiation of inflammaging [87, 88]. In aged vascular smooth muscle cells (VSMCs), TLR4 activation leads to the production of pro-inflammatory cytokines such as IL-1, IL-6, and IL-8, while concurrently inhibiting the expression of anti-inflammatory factors, thus disrupting immune homeostasis [89]. Microorganisms infiltrating aging epithelial barriers collaborate with pattern recognition receptors (PRRs), giving rise to the production of cytokines, which results in the accumulation of myeloid-biased hematopoietic stem cells (HSCs). Myeloid-biased HSCs contribute to inflammaging by elevating the secretion of IL-1 and TLR4 ligands into the bloodstream [90]. TLR4 activity in the gingival region inhibits B cell-specific Moloney murine leukemia virus integration site 1 (Bmi-1), enhancing the NLRP3 inflammasome and senescence-associated secretory phenotype (SASP). This cascade of events actively participates in the aging process [91]. These findings collectively underscore the notion that TLR4 activation during the aging process precipitates a chronic inflammatory response, instigating inflammaging, and in turn, exerting a profound influence on organismal aging, with far-reaching implications for the prospects of enduring health.

## TLR4 and aging-related disease

TLR4 has been reported to be associated with various aging-related diseases. Prolonged exposure to chronic inflammatory responses resulting from TLR4 overexpression or hyperactivation can influence the onset and progression of such diseases. Conversely, the inhibition of TLR4 may also contribute to disease pathogenesis. Consequently, the modulation of TLR4 expression or the regulation of its downstream signaling pathways is emerging as a promising therapeutic approach for addressing age-related diseases. In this review, we aim to consolidate the multifaceted role of TLR4 in diverse age-related diseases and underscore its potential as a therapeutic target (see Table 1).

**Table 1:** TLR4 regulates age-related diseases

Organ	Control factor	Signaling pathway	Disease and symptom	Reference
Brain	DL0410	TLR4/MyD88/NF-κB signaling	Alzheimer’s disease BBB protection	[92]
	Tea polyphenol	TLR4/NF-κB signaling	Memory loss defense	[93]
	Naringin	TLR4/NF-κB signaling	Hippocampus recovery	[94]
	CAY10614 (TLR4 antagonist)	LPS/TLR4 signaling	Hippocampal cell senescence inhibition	[95]
	Probiotic combination	TLR4/NF-κB signaling	Hippocampus defense	[96]
	TLR4 antagonist	HMGB1/TLR4 signaling	Perioperative neurocognitive disorders (PNDs) recovery	[97]
	TDZD-8 (GSK-3β inhibitor)	TLR4 expression	BBB protection	[98]
Heart		HMGB1/TLR2, 4 signaling	Cardiac tissue aging	[99]
	HSP70	TLR4/NF-κB signaling	Deterioration of heart function (global I/R)	[100]
	Palmitate	TLR4, NLRP3 inflammasome	Cardiac fibroblasts progression	[101]
	NcoR1, HDAC1	TLR4/autophagy	Cardiac remodeling and contractile dysfunction	[102]
		TLR4 expression	Endothelial-dependent vascular relaxation inhibition	[103]
	Klotho	TLR4 signaling	Promoted differentiation of M2a/M2c macrophages improved myocardial viability	[104]
Adipose tissue	Fet A	TLR4 deficiency	Decreased inflammation Increased glucose tolerance	[105]
Pancreatic island		TLR4 deficiency	Decreased inflammation Protect insulin homeostasis	[106]
Adipocyte	SPARC	TLR4/IRF3 signaling	Increased inflammation protects metabolic function	[107]
Muscle		LPS/TLR4 signaling	Metabolic endotoxemia induction	[108]
Cartilage	DAP12	TLR4 signaling	Cartilage catabolism	[109]
	PUM1	TLR4/NF-κB signaling	Chondrogenic potential of MSCs improvement	[110]
Lung		TLR4 expression	Alveoli collapse Lung volume expansion	[111]
	HDAC2/p16^INK4a^	TLR4 expression	Endothelial cell senescence Alveoli collapse Lung volume expansion	[112]
Blood	Lipoteichoic acid, Pam3CysK	TLR4/ TNF-α signaling	Sepsis inducement	[113]
Myeloma cell	DSUP/p53, p16	TLR4 signaling	Myeloma cell senescence	[114]
Placenta	Preeclampsia (PE)	TLR4, HH signaling	Placenta development, PMSC senescence	[115]
Bone marrow stromal cell	S100A9	TLR4 signaling, NLRP3 inflammasome	Cell senescence induction	[116]
Intestinal mucosa	Wheat oligopeptides	TLR4/Myd88/MAPK signaling	Intestinal barrier protection	[117]
	Alginate oligosaccharide	TLR4/NF-κB signaling	Mucosal epithelial cell senescence, IMB maintainence	[118]
Skin	miR-326-3p	TLR4 expression	Skin cell senescence induction	[119]
Macrophage	cyto OH	OxPL/TLR4 signaling	Macrophage activation, Apoptosis promotion	[120]
Dental pulp cell	TLR4 neutralizing antibodies and inhibitor	TLR4/NF-κB signaling	Cell senescence inhibition	[121]

### Brain disease

Aged rats are known to develop Alzheimer’s disease as a consequence of oxidative damage and neuroinflammation [92]. When subjected to DL0410 treatment in a D-galactose (D-gal)-induced aging rat model, improvements were observed in learning and cognitive function, alongside the protection of blood-brain barrier (BBB) integrity. DL0410 exerted its beneficial effects by promoting an antioxidative response and mitigating the production of inflammatory cytokines such as TNF-α, IL-1β, and IL-6. This favorable outcome was attributed to the inhibition of the TLR4/MyD88/TRAF6/NF-κB signaling pathway in LPS-stimulated BV2 cells.

Aging rats commonly experience memory loss, which has been linked to increased intestinal epithelial permeability due to dysbiosis of the intestinal flora and activation of the TLR4/NF-κB signaling pathway within the hippocampus [93]. Treatment with tea polyphenols has been shown to rectify intestinal flora dysbiosis, subsequently modulating the TLR4-mediated inflammatory signaling pathway in the hippocampus, ultimately protecting against memory loss.

Naringin alleviates histopathological damage found in the hippocampus of rats treated with D-gal, a senescence inducer [94]. The recovery of the hippocampus by Naringin is dependent on the decreased activity of the TLR4/NF-κB signaling, which relieves the inflammatory response, oxidative stress, and ER stress while increasing the neurotrophic factors brain-derived neurotrophic factor (BDNF) and nerve growth factor (NGF). Long-term culture of rat hippocampal neurons promotes cellular senescence and increases cell death when exposed to LPS, but this phenomenon is not observed when cultured for a short-term [95]. The expression of TLR4 is increased in aged hippocampal cells, and cell senescence decreases with treatment of the TLR4 antagonist CAY10614.

A promising anti-aging solution involving a probiotic combination has shown efficacy in mitigating age-related cognitive decline, motor dysfunction, and impaired exploratory behavior in the senescence accelerated mouse-prone 8 (SAMP8) model [96]. This probiotic combination not only reduced TLR4/NF-κB signaling activity, which triggers brain inflammatory responses, but also increased the expression of Sirt1 for hippocampal defense. Additionally, it improved intestinal microbiota composition and increased levels of intestinal permeability-related proteins.

In older rats, perioperative neurocognitive disorders (PNDs) associated with morphine administration persist due to TLR4-mediated neuroinflammation [97]. T Treatment with a TLR4 antagonist from the bacterium Rhodobacter sphaeroides improves memory loss caused by morphine administration in aged rats, mitigates the dysregulation of postsynaptic proteins, and preserves mature BDNF levels. Notably, TLR4 antagonists appear to improve PNDs by blocking TLR4 activation by the endogenous ligand HMGB1, rather than by directly blocking morphine or its metabolites. Furthermore, abnormal BDNF permeability and cognitive impairment were observed in older mice, correlated with increased TLR4 expression and decreased phosphorylation of serine 9, an inhibitory residue in GSK-3β protein [98]. Treatment with a GSK-3β inhibitor, 4-benzyl-2-methyl-1,2,4-thiadiazolidine-3,5-dione (TDZD-8), ameliorated cognitive impairment by reducing TLR4 expression and increasing the expression of junction proteins (claudin1 and claudin5) in endothelial cells of the BBB.

### Cardiac disease

To investigate the etiology of aging-related heart disease, a comparative analysis was conducted on the hearts of senescence-resistant control (SAMR1) mice and SAMP8 mice at 6 months of age [99]. The study revealed that the expression of HMGB1, TLR2, TLR4, and the secretion of pro-inflammatory cytokines (such as IFN, IL-1, and IL-6) in the cardiac tissue of the SAMP8 mice were increased. Furthermore, a decrease in the marker protein of M2 macrophages (associated with an anti-inflammatory phenotype) in SAMP8 mice indicated that HMGB1-TLR2/4 signaling plays a significant role in age-related heart disease. In a comparison of the hearts of aging (18–24 months) and adult (4–6 months) mice subjected to global ischemia/reperfusion (I/R), it was observed that the expression of HSP27 and cytokines significantly increased in the hearts of aging mice [100]. Additionally, the expression of TLR4 was markedly elevated in the aging mouse hearts, and TLR4/NF-κB signaling was activated in response to I/R or HSP27 stimulation. Inhibiting HSP27 proved effective in reducing inflammatory reactions and restoring the function of the aging heart.

Palmitate causes inflammatory reactions through TLR4 and NLRP3 inflammasome activation in cardiac fibroblasts and ROS formation in mitochondria, leading to mitochondrial dysfunction [101]. Palmitate also induces the characteristic phenotype of cellular senescence, inhibiting cell proliferation, increasing the expression of matrix metalloproteinase-2 (MMP-2), and elevating the activity of senescence-associated β-galactosidase (SA-β-gal) through NLRP3-independent pathways. These findings suggest the potential for myocardial disease development via cellular senescence. In the hearts of aging mice, autophagy is diminished, but in TLR4-deficient (-/-) mice, myocardial function and autophagy are enhanced compared to wild-type (WT) mice [102]. The downregulation of nuclear receptor corepressor 1 (NCoR1) and histone deacetylase 1 (HDAC1) expression in aging mice is reversed in TLR4-/- mice, suggesting a pivotal role of TLR4 ablation in regulating cardiac remodeling, contractile dysfunction, and autophagy during aging. Cardiac remodeling and contractile dysfunction are common manifestations of the aging process, and they significantly contribute to the onset and progression of cardiac disorders and diseases. The upregulation of TLR4 expression with aging was observed in both mouse heart and aortic tissues [103]. TLR4 deficiency led to a reduction in the secretion of pro-inflammatory cytokines within the heart and aorta, subsequently resulting in enhanced cardiac function and endothelial-dependent vascular relaxation in aged mice. These findings suggests the potential of TLR4 as a therapeutic target for age-related cardiovascular diseases.

Klotho, an anti-aging protein, functions to protect heart aging from the D-galactosamine-induced cardiac senescence [104]. Soluble Klotho promoted the differentiation of M2a/M2c macrophages and enhanced myocardial viability by protecting cardiomyocytes from senescence through the inhibition of TLR4 signaling in RAW264.7 cells. The improvement in aging myocardial function and viability sends a positive signal for the novel therapy of age-related heart disease.

### Diabetes

Aging is one of the main causes of diabetes, and understanding the role of TLR4 signaling and pro-inflammatory factors in the aging process may propose new therapeutic targets. Unlike dietary-induced obesity model mice, the expression of FetA and TLR4 in adipose tissue was normal in aging mice [105]. However, the reduced inflammation observed in adipose tissue and improved glucose tolerance in aging TLR4-deficient mice suggest a potential connection between TLR4 and the inflammatory response induced by aging in adipose tissue. Prolonged and low-level inflammation can lead to insulin resistance and the impairment of β-cells, ultimately contributing to the development of type 2 diabetes [106]. Aged mice placed on a high-fat diet (HFD) exhibited significantly impaired glucose tolerance and increased tissue inflammation compared to young mice on an HFD, but this phenomenon was attenuated in TLR4-deficient mice. Inhibition of TLR4 reduced the secretion of inflammatory cytokines and the differentiation of pro-inflammatory macrophages, safeguarding insulin secretion and homeostasis within the pancreatic islets, particularly under the influence of an HFD and aging.

Secreted protein acidic and rich in cysteine (SPARC) functions to convert anti-inflammatory macrophages to pro-inflammatory ones via interferon-stimulated gene (ISG) induction [107]. Inhibition of SPARC in adipocytes mitigates the age-induced inflammatory response, thus preserving metabolic function. These SPARC-related functions are regulated by TLR4-mediated TBK1, IRF3, IFN-β, and STAT1 signaling pathways.

Alterations in glucose metabolism and the development of sarcopenia due to aging contribute to the onset of type 2 diabetes [108]. In older individuals, there is a notable increase in plasma levels of the TLR4 agonist LPS and its associated binding protein, indicating the presence of metabolic endotoxemia. While an aerobic exercise program enhances insulin sensitivity, it appears to be influenced by metabolic endotoxinosis, as it does not seem to have any effect on muscle TLR4, NF-κB, and JUK.

### Osteoarthritis

DNAX-activating protein of 12kDA (DAP12) is an adapter protein that limits the activity of TLR4 [109]. When a high-fat diet (HFD) was administered to female mice in the WT, TLR4 knock-out (KO), and DAP12 KO groups, only the TLR4 KO mice did not exhibit cartilage catabolism. These findings suggest that TLR4 promotes HFD-induced cartilage degradation in aging female mice, whereas DAP12 restricts it.

PUM1 binds to the 3’-UTR of TLR4, leading to a reduction in TLR4 mRNA levels and the inhibition of NF-κB activity, ultimately suppressing the cellular senescence of human mesenchymal stem cells (MSCs) induced by H_2_O_2_ [110]. Furthermore, the inhibition of TLR4/NF-κB activity by PUM1 improved the chondrogenic potential of MSCs and provided protection against inflammation-mediated disruption of chondrogenic formation in osteoarthritis mouse models.

### Emphysema

A typical symptom of emphysema is the collapse of the alveolar wall and an increase in lung size, resulting in decreased blood oxygen saturation [122]. In comparison to WT mice, it was observed that emphysema symptoms manifested in an age-dependent manner without external stimulation in TLR4-deficient mice [111]. In mice older than 3 months, alveolar walls collapsed, lung volume increased, and chord length elongated, all indicative of emphysema. These emphysema symptoms are notably influenced by the level of TLR4 expression in lung endothelial cells [112]. Lung endothelial cells in TLR4-deficient mice exhibited accelerated aging compared to those in WT mice, following a passage-dependent pattern. This was accompanied by a significant increase in the mRNA and protein levels of p16^INK4a^, a protein associated with aging. The expression of p16^INK4a^ was regulated by the acetylation of Histone H4 lysine 8 residue due to reduced levels of HDAC2. Although the downstream signaling of TLR4 has not been precisely identified, it has been confirmed that alterations in TLR4 expression are pivotal in the development of emphysema.

### Other diseases

TNF-α is widely recognized as a critical mediator in sepsis, and TLR4 plays a central role in initiating signaling pathways that lead to TNF-α production [113]. Through whole blood assays, it was observed that TNF-α levels increased with aging, while the responses to stimulation by LPS, lipoteichoic acid, and Pam3CysK decreased. Aging leads to elevated baseline TNF-α production by circulating leukocytes and impaired responses to TLR2 and TLR4 agonists, potentially rendering older adults more susceptible to sepsis.

When H_2_O_2_ induces cell senescence in the myeloma cell line RPMI8226, TLR4 activity decreases, accompanied by a reduction in the expression of dual-specificity phosphatases (DUSP), while the aging marker proteins p16^INK4A^ and p53 increase [114]. The inhibition of DUSP represses H_2_O_2_-induced senescence in myeloma cells, whereas overexpression of DUSP or inhibition of TLR4 facilitates senescence.

Preeclampsia (PE) is a pregnancy disorder influenced by placental aging, and placental mesenchymal stem cells (PMSCs) play a role in controlling placental development and senescence [115]. Activation of TLR4 by LPS accelerates the senescence of PMSCs by inhibiting the Hedgehog (HH) pathway, both in vivo and in vitro. This is associated with detrimental paracrine effects that affect uterine spiral artery remodeling and placental angiogenesis.

Bone marrow stromal cells in patients with myelodysplastic syndrome (MDS) exhibit aging characteristics, and overexpression of S100A9 has been identified as an inducer of aging in low-risk MDS patients [116]. Exogenous S100A9 treatment promotes senescence in bone marrow stromal cells and human stromal cells, but this senescence is blocked by the inhibition of TLR4. Since S100A9-induced cell senescence promotes the formation of the NLRP3 inflammasome and the production of IL-1β, it can be inferred that S100A9 regulates bone marrow stromal cell senescence through TLR4 and the NLRP3 inflammasome.

Wheat oligopeptides (WP) pretreatment enhances intestinal mucosal protection by exerting antioxidant activity, reducing malondialdehyde levels in the small intestine mucosa [117]. WP also suppresses the secretion of pro-inflammatory cytokines, such as TNF-α, TGF-β, IFN-γ, IL-1β, and IL-6, attenuating the inflammatory response and upregulating the expression of junction proteins, including zonula occludens-1 (ZO-1) and junctional adhesion molecule-A (JAM-A), to safeguard the intestinal mucosa. WP functions to mitigate the inflammatory response associated with aging and protect the intestinal barrier by inhibiting the TLR4/MyD88/MAPK signaling pathway.

Alginate oligosaccharide (AOS) maintains the function of the intestinal mucosal barrier (IMB) by enhancing permeability and increasing the expression of tight junction proteins in aging mice and NCM460 cells [118]. AOS achieves this by blocking the TLR4/NF-κB pathway while enhancing the expression of Fibroblast growth factor 1 (FGF1). In the aging mechanism of skin fibroblasts, miR-326-3p inhibits the expression of TLR4, thereby promoting cellular senescence [119]. Conflicting correlations between miR-326-3p and TLR4 are observed in the aging skin tissue of mice.

Cytoplasmic hydroxyl radicals (cyto OH), inducers of cell senescence, promoted the production of oxidized phospholipids (Ox-PLs), which act as ligands for TLR4. This induction subsequently activated macrophages and exacerbated apoptosis, inflammation, and fibrosis [120].

Treatment with TLR4 neutralizing antibodies and a TLR4 inhibitor effectively inhibited excessive ROS production, DNA telomere damage, as well as the production of inflammatory cytokines including IL-1β, IL-6, IL-8, and TNF-α. Additionally, it suppressed NF-κB/MAPK activation and significantly attenuated dental pulp cell senescence [121].

## Conclusion

TLR4, a receptor with a central role in innate immune signaling, orchestrates inflammatory responses by modulating the activity of transcription factors. Recent research has unveiled the significance of regulating TLR4 activity and expression in the context of age-related diseases. This paper provides an overview of how TLR4 governs cellular senescence across diverse tissues, including the brain, heart, lungs, and joints, and contributes to the onset and progression of age-related disorders.

Alterations in TLR4 activity exert control over NF-κB and MAPK activation, employing both MyD88-dependent and independent pathways. Consequently, this modulation influences the severity of various ailments such as Alzheimer’s disease, myocardial disorders, and diabetes by impacting the expression of pro-inflammatory cytokines like IL-1β, IL-6, TNF-α, and type 1 interferons. Age-related variations in TLR4 expression have been identified, with functional implications in managing conditions like emphysema, osteoarthritis, and cardiovascular resilience. These findings shed light on a novel role for TLR4 in the treatment of age-related diseases.

Continued exploration of the influence of TLR4 and the specific mechanisms operating in each tissue promises valuable insights into therapeutic strategies for age-related diseases.

## Data Availability

Not applicable.
